# Patterns of Bone Mineral Density Loss at Multiple Skeletal Sites Following Recent Menopause in Users and Non-Users of Menopausal Hormone Therapy

**DOI:** 10.1007/s00223-025-01392-8

**Published:** 2025-06-04

**Authors:** Kara L. Holloway-Kew, Amelia G. Morse, Kara B. Anderson, Mark A. Kotowicz, Julie A. Pasco

**Affiliations:** 1 Deakin University, IMPACT – the Institute for Mental and Physical Health and Clinical Translation, School of Medicine, Geelong, Australia; 2 Department of Medicine - Western Health, The University of Melbourne, St Albans, Australia; 3 University Hospital Geelong, Barwon Health, Geelong, Australia; 4 School of Public Health and Preventive Medicine, Monash University, Prahran, Australia

**Keywords:** Bone mineral density, Menopause, Osteopenia, Osteoporosis

## Abstract

Post-menopausal bone loss has been well described, however fewer studies have focussed on changes around the time of menopause. This study describes bone mineral density (BMD) loss following recent menopause, stratified by hormone replacement therapy (HT) use. Women (n = 287) who self-reported recent menopause (≥ 12 months to < 5 yr since last menstrual period) for at least one assessment phase of the Geelong Osteoporosis Study were included. BMD was measured using Lunar DPX-L and GE-Prodigy machines. Time since menopause was calculated for each participant at each assessment phase and divided into three categories: < 5 yr, 5-10 yr and ≥ 10 yr. BMD loss was expressed as: (i) cumulative loss over time, (ii) absolute value per year and (iii) percentage loss per year. Proportions of women with normal BMD, osteopenia and osteoporosis were also calculated. Cumulative BMD loss was lower among HT users than non-users at all sites and time categories, except the femoral neck. Compared to the other time categories, HT non-users had a greater rate of BMD loss (expressed as an absolute value or percentage per year) during the first five years postmenopause at the ultra-distal forearm and lumbar spine. No differences were observed between the time categories for HT users. The proportions of women with osteopenia and osteoporosis increased across each of the time categories, but patterns differed by skeletal site, being more pronounced for the femoral neck and mid-forearm sites. Rates of bone loss were greater at the lumbar spine and ultra-distal forearm during the first five years following menopause.

**Supplementary Information:**

The online version contains supplementary material available at 10.1007/s00223-025-01392-8.

## Introduction

Postmenopausal bone loss and the consequent increase in fracture risk has been clearly described, with one in three women aged ≥ 50 years experiencing a fracture in their remaining lifetime [1, 2]. Fewer studies have focused on changes in bone around the time of menopause.

It has previously been reported that bone loss begins during the menopause transition, rather than in the years after menopause has occurred. For example, the Study of Women’s Health Across the Nation (SWAN) followed 862 women over a ten-year period; five years prior to and five years following the final menstrual period (FMP) [3]. The study showed that the greatest loss bone mineral density (BMD) at the femoral neck and lumbar spine occurred one year before and two years after the FMP. Another study that followed a total of 199 monozygotic and 125 dizygotic twin pairs (age 25–75 years) over 1.5 to 4.5 years has also reported similar results [4]. Using high resolution peripheral quantitative computed tomography (HR-pQCT), the study showed that no significant bone loss was observed prior to menopause. The largest amount of bone loss occurred during the transition to menopause, and primarily at cortical (80%) rather than trabecular sites (20%).

Data from the SWAN study has also shown that changes in bone turnover begin to increase approximately two years prior to the FMP; then plateau around 1.5 years after the FMP [5]. This increase in bone turnover and rate of bone loss around the time of menopause have also been associated with an increased risk of incident fracture [6, 7]. Additionally, the age at menopause has been shown to be an important risk factor for future fracture [8]. In another SWAN study, a total of 1554 women aged 42 to 52 years at study baseline were followed for a mean duration of 22 years. Women who experienced menopause at age 47 years had a 23% and 34% higher fracture risk than women who experienced menopause at 52 and 55 years, respectively.

It is important to understand the nature of bone loss during and shortly after menopause because it can affect future fracture risk, as well as assist in the selection of effective anti-fracture therapies [9]. Therefore, this study aimed to examine changes in BMD over time among women who transitioned through menopause during a follow-up assessment phase of the Geelong Osteoporosis Study.

## Methods

### Participants

Participants for this study were women from the Geelong Osteoporosis Study (GOS), a longitudinal study that recruited residents from the Barwon Statistical Division using a randomised, age-stratified sampling method. Further details of the study are provided elsewhere [10]. Participants were recruited at baseline (1993–1997) and returned for follow-up assessments at 2 yr (1995–2001), 4 yr (1998–2002), 6 yr (2000–2008), 8 yr (2001–2006), 10 yr (2004–2008) and 15 yr (2011–2014) follow-up phases. Participants were included in this study if they reported recent (< 5 years) menopause during at least one of the GOS study assessment phases (n = 295). If a participant reported that menopause had occurred ≥ 5 years prior to all assessment phases completed, they were excluded.

### Menopause Status

Menopause status was self-reported at all assessment phases. Recent menopause was considered if a participant reported it had been ≥ 12 months to < 5 yr since their last menstrual period. For participants who had reported recent menopause for at least one GOS assessment phase, the time since menopause was calculated for each assessment phase where they had participated. Age at menopause was also determined for each participant.

### Bone Mineral Density Measurements

BMD was measured using a Lunar DPX-L (Lunar; Madison, WI, USA) dual-energy X-ray absorptiometry (DXA) machine from baseline to 10 yr follow-up. When this scanner became outmoded, a GE-Prodigy (Prodigy; GE Lunar, Madison, WI, USA) was used for the 15 yr follow-up. Cross-calibration of the densitometers revealed no mean differences in lumbar spine or femoral neck BMD for 40 individuals (age 21-82 yr) [10]. BMD was measured at four skeletal sites for each assessment phase: femoral neck, lumbar spine, ultra-distal forearm and mid-forearm.

### Other Measurements

Weight and height were measured to the nearest 0.1 kg and 0.1 cm, respectively, using electronic scales and a wall-mounted Harpenden stadiometer. Body mass index (BMI) was calculated as weight(kg)/(height in m)^2^. Fractures prior to menopause were self-reported and confirmed using radiological reports where possible. Smoking status and alcohol consumption were self-reported. Participants were considered current smokers or not. High alcohol consumption was categorised as ≥ 20 g or 2 standard drinks per day. Physical activity was self-reported in seven categories including very active, active, sedentary, limited, inactive, chair/bedridden and bedfast. This was dichotomised as “high” physical activity including very active and active, while the remaining responses were categorised as “low” physical activity. Participants were also asked to self-report hormone replacement therapy (HT) use at each assessment phase. Participants using bisphosphonates were excluded from analyses (n = 8), and no participants were taking any other type of anti-fracture therapy.

### Statistical Analyses

Continuous variables were presented using mean ± SD or median (IQR) where appropriate. Differences between women who did and did not use HT were determined using t tests or Mann–Whitney tests for continuous variables. Categorical variables were presented as n(%) and differences determined using a Chi-square test.

Comparisons of BMD loss were made across three different time categories since menopause: < 5 yr, 5 to < 10 yr and ≥ 10 yr. The amount of BMD lost was calculated for each participant at the four skeletal sites and expressed as (i) a cumulative BMD loss over time (%), (ii) an absolute value per year (g/cm^2^) and (iii) a percentage loss per year (%). Comparisons of BMD loss across the three different time categories since menopause were completed using ANOVA. Each participant contributed to one or more of these time categories, depending upon how many follow-ups they attended where BMD was measured. These analyses included 208 women who had at least two measurements of BMD. Analyses were stratified by HT use, which was updated at each follow-up phase, and therefore could be different for a participant across the different menopause time categories.

Additionally, BMD categories were determined at all four skeletal sites for each participant at each assessment phase where they had BMD measured. For each participant, BMD values were converted to T scores using Australian reference ranges [11, 12]. BMD categories included normal (T score ≥ -1.0), osteopenia (T score < -1.0 and ≥ -2.5) and osteoporosis (T score < -2.5). Comparisons between groups were completed using a Chi-square test. All 295 women had at least one measurement of BMD and contributed to this analysis.

Analyses were completed using Stata (Version 17. Stata Corp. 2017. Stata Statistical Software: Release 17. College Station, TX: StataCorp LLC).

## Results

After exclusion of eight participants who had used bisphosphonates, there were 287 women who reported recent menopause during one of the assessment phases, and were included in the analyses. Of these, 76 (26.5%) reported recent menopause at baseline, 28 (9.8%) at 2 yr, 28 (9.8%) at 4 yr, 50 (17.4%) at 6 yr, 13 (4.5%) at 8 yr, 35 (12.2%) at 10 yr and 57 (19.9%) at 15 yr.

Table 1 shows the descriptive characteristics of the participants included in this study. Overall, women were in the overweight category for BMI and approximately one in ten had sustained a prior fracture. T scores for BMD at all four skeletal sites were in the normal range (≥ -1.0). HT use around the time of menopause was reported by one in five women, mainly at the first few assessment phases following the report of menopause. The median time since menopause across all visits for HT users was 5.0 (2.7, 7.9) years. Women who were non-users of HT at menopause had greater weight and BMI than women who did use HT (Table 1). No other differences were observed between those who did and did not use HT around the time of menopause.

**Table 1:** Descriptive characteristics of the women included in this study (n = 287), stratified by hormone replacement therapy (HT) status. Data from the assessment phase at which menopause was first identified. Data presented as mean ± SD, median(IQR) or n(%) as appropriate

	All participants (n = 287)	HT non-users (n = 230)	HT users (n = 57)	p value
Age (yr)	54 (50, 56)	54 (50, 56)	53 (51, 56)	0.754
Age at menopause (yr)	51 (48, 53)	51 (48, 53)	51 (49, 53)	0.466
Weight (kg)	72.9 ± 15.5	73.9 ± 15.6	68.8 ± 14.3	**0.020**
Height (cm)	162.0 ± 5.9	162.1 ± 5.7	161.5 ± 6.4	0.542
Body mass index (kg/m^2^)	27.8 ± 5.9	28.2 ± 5.9	26.4 ± 5.3	**0.030**
Prior fracture	27 (9.4)	24 (10.4)	3 (5.3)	0.247
High alcohol consumption	35 (12.2)	27 (11.7)	8 (14.0)	0.313
Smoking status	49 (17.1)	38 (16.5)	11 (19.3)	0.597
Physical activity	57 (19.9)	48 (20.9)	9 (15.8)	0.412
Femoral neck BMD (g/cm^2^)	0.946 ± 0.140	0.948 ± 0.136	0.937 ± 0.156	0.629
Femoral neck BMD T score	− 0.569 ± 1.100	− 0.551 ± 1.069	− 0.640 ± 1.232	0.629
Lumbar spine BMD (g/cm^2^)	1.212 ± 0.170	1.210 ± 0.165	1.220 ± 0.191	0.722
Lumbar spine BMD T score	− 0.154 ± 1.258	− 0.168 ± 1.212	− 0.094 ± 1.418	0.722
Ultra-distal forearm BMD (g/cm^2^)	0.335 ± 0.053	0.337 ± 0.053	0.325 ± 0.055	0.154
Ultra-distal forearm T score	0.190 ± 1.182	0.241 ± 1.168	-0.025 ± 1.229	0.154
Mid-forearm BMD (g/cm^2^)	0.712 ± 0.061	0.713 ± 0.059	0.708 ± 0.068	0.645
Mid-forearm BMD T score	0.235 ± 1.092	0.251 ± 1.061	0.168 ± 1.221	0.645

Bold values indicate a statistically significant difference between groups

*BMD* Bone mineral density

Missing data: weight, height, body mass index, n = 4; prior fracture n = 5; alcohol consumption n = 51; smoking status n = 3; physical activity n = 2; femoral neck BMD n = 7; lumbar spine BMD n = 4; ultra-distal BMD and mid-forearm BMD, n = 8

### Bone Mineral Density Loss

Table 2 and Supplementary Fig. 1 show the cumulative BMD loss (%) versus time since menopause for each of the skeletal sites. There were no differences between the sites for the time category < 5 yr (p > 0.05). However, for the time category 5 to < 10 yr, BMD loss at the ultra-distal forearm was higher than at the other sites (p < 0.001) for HT users and non-users. For the time category ≥ 10 yr, among HT non-users, BMD loss at the lumbar spine was less pronounced than at the other sites. Among HT users, BMD loss was greater at the femoral neck compared to the other sites. For all sites except the femoral neck, the percentage BMD loss was lower for HT users compared to non-users (Table 2).

**Table 2:** Cumulative bone mineral density (BMD) loss for time intervals since menopause of < 5 yr, 5 to < 10 yr and ≥ 10 yr. Data stratified by hormone replacement therapy (HT) use

	Femoral neck			Lumbar spine			Ultra-distal forearm			Mid-forearm			
HT non-users	N	Age^a^ (yr)	% BMD loss (95%CI)	N	Age^a^ (yr)	% BMD loss (95%CI)	N	Age^a^ (yr)	% BMD loss (95%CI)	N	Age^a^ (yr)	% BMD loss (95%CI)	p value^b^
< 5 yr	82	54.8 (51.9–57.2)	− 1.33 (− 2.45, − 0.21)	80	54.8 (51.9–57.1)	− 2.29 (− 3.43, − 1.16)	82	54.8 (51.9–57.2)	− 3.12 (− 4.24, − 2.00)	82	54.8 (51.9–57.2)	− 1.22 (− 2.34, − 0.10)	0.061
5 to < 10 yr	216	57.9 (54.7–60.1)	− 2.39 (− 3.33, − 1.45)	215	57.9 (54.6–60.1)	− 1.93 (− 2.87, − 0.99)	216	58.0 (54.7–60.1)	− 4.71 (− 5.65, − 3.77)	216	58.0 (54.7–60.1)	− 2.03 (− 2.97, − 1.09)	** < 0.001**
≥ 10 yr	203	64.2 (60.9–67.2)	− 7.70 (− 9.00, − 6.40)	207	64.2 (60.9–67.2)	− 4.38 (− 5.67, − 3.09)	209	64.2 (60.9–67.2)	− 6.07 (− 7.35, − 4.79)	209	64.2 (60.9–67.2)	− 6.92 (− 8.20, − 5.64)	**0.003**
HT users	N	Age^a^ (yr)	% BMD loss (95%CI)	N	Age^a^ (yr)	% BMD loss (95%CI)	N	Age^a^ (yr)	% BMD loss (95%CI)	N	Age^a^ (yr)	% BMD loss (95%CI)	p value^b^
< 5 yr	33	54.1 (49.9–56.7)	− 0.00 (− 1.62, 1.61)	33	54.1 (49.9–56.7)	0.14 (− 1.47, 1.76)	33	54.1 (49.9–56.7)	− 0.69 (− 2.30, 0.93)	33	54.1 (49.9–56.7)	0.83 (− 0.78, 2.45)	0.628
5 to < 10 yr	75	56.6 (52.3–59.2)	− 1.41 (− 2.80, − 0.01)	76	56.4 (52.3–59.1)	0.64 (− 0.74, 2.03)	74	56.4 (52.3–58.9)	− 2.87 (− 4.28, − 1.47)	74	56.4 (52.3–58.9)	0.86 (− 0.55, 2.27)	** < 0.001**
≥ 10 yr	27	61.3 (58.9–63.9)	− 6.45 (− 9.29, − 3.61)	27	61.3 (58.9–63.9)	1.08 (− 1.76, 3.91)	26	61.2 (58.9–63.9)	− 1.50 (− 4.40, 1.39)	26	61.2 (58.9–63.9)	− 1.08 (− 3.97, 1.82)	**0.003**
HT users vs non-users			p value^c^			p value^c^			p value^c^			p value^c^	
< 5 yr			0.248			**0.010**			**0.038**			**0.006**	
5 to < 10 yr			0.339			** < 0.001**			**0.039**			** < 0.001**	
≥ 10 yr			0.425			**0.002**			**0.010**			** < 0.001**	

Bold values indicate a statistically significant difference between groups

^a^ Age presented as median (interquartile range)

^b^ p value comparing the four skeletal sites across each time interval

^c^ p value comparing cumulative BMD loss between HT users and non-users at each site and time interval

Table 3 shows the results for rate of BMD lost per year (expressed as an absolute value or percentage) for each of the time categories. For HT non-users, BMD loss expressed as an absolute value was greater during the < 5 yr time category compared to the other two time categories for both the lumbar spine and ultra-distal forearm. At the mid-forearm, the absolute value for BMD loss was greater during the < 5 yr category and ≥ 10 yr category compared to the 5 to < 10 yr category. When considering BMD loss as a percentage decrease per year, the results were similar (Table 3).

**Table 3:** Bone mineral density (BMD) loss per year, expressed as absolute value or percentage for time intervals since menopause of < 5 yr, 5 to < 10 yr and ≥ 10 yr. Data stratified by hormone replacement therapy (HT) use

	< 5 yr			5 to < 10 yr			≥ 10 yr			
Value per year (g/cm^2^)	N	Age^a^ (yr)	Mean BMD loss (95%CI)	N	Age^a^ (yr)	Mean BMD loss (95%CI)	N	Age^a^ (yr)	Mean BMD loss (95%CI)	p value^b^
HT non-users										
Femoral neck	137	56.3 (52.9–58.6)	− 0.007 (− 0.010, − 0.003)	89	60.4 (57.8–63.6)	− 0.008 (− 0.012, − 0.003)	36	70.2 (66.5–72.2)	− 0.009 (− 0.016, − 0.002)	0.872
Lumbar spine	137	56.4 (52.9–58.8)	− 0.011 (− 0.015, − 0.008)	89	60.5 (57.8–63.6)	− 0.003 (− 0.007, − 0.000)	35	69.7 (65.1–72.2)	− 0.006 (− 0.012, 0.000)	0.005
Ultra-distal forearm	138	56.5 (53.0–58.9)	− 0.004 (− 0.006, − 0.003)	92	60.4 (57.7–63.7)	− 0.000 (− 0.002, 0.002)	38	70.2 (66.3–72.1)	0.002 (− 0.002, 0.005)	0.001
Mid-forearm	139	56.5 (53.1–59.0)	− 0.005 (− 0.007, − 0.003)	90	60.4 (57.7–63.5)	− 0.001 (− 0.004, 0.002)	38	70.2 (66.3–72.1)	− 0.008 (− 0.013, − 0.004)	0.013
HT users										
Femoral neck	67	55.0 (51.6–58.0)	− 0.001 (− 0.007, 0.004)	50	58.8 (54.4–61.9)	− 0.006 (− 0.012, 0.000)	12	67.7 (66.2–71.6)	− 0.011 (− 0.024, 0.002)	0.277
Lumbar spine	68	55.1 (51.7–57.9)	− 0.003 (− 0.008, 0.002)	50	58.8 (54.4–61.9)	− 0.002 (− 0.007, 0.004)	13	68.0 (66.3–71.8)	− 0.008 (− 0.020, 0.003)	0.569
Ultra-distal forearm	67	55.0 (51.6–58.0)	− 0.002 (− 0.004, − 0.000)	49	58.8 (54.3–62.0)	− 0.002 (− 0.004, 0.001)	12	67.7 (61.8–71.6)	− 0.001 (− 0.005, 0.004)	0.807
Mid-forearm	67	55.0 (51.6–58.0)	0.000 (− 0.003, 0.004)	50	58.8 (54.4–62.1)	0.000 (− 0.004, 0.004)	12	67.7 (66.2–71.6)	− 0.008 (− 0.017, 0.000)	0.183
			< 5 yr			5 to < 10 yr			≥ 10 yr	
Percentage per year (%)	N	Age^c^ (yr)	Mean BMD loss (95%CI)	N	Age^c^ (yr)	Mean BMD loss (95%CI)	N	Age^c^ (yr)	Mean BMD loss (95%CI)	p value^a^
HT non-users										
Femoral neck	137	-	− 0.683 (− 1.063, − 0.302)	89	-	− 0.796 (− 1.268, − 0.325)	36	-	− 0.864 (− 1.606, − 0.122)	0.882
Lumbar spine	137	-	− 0.909 (− 1.165, − 0.654)	89	-	− 0.246 (− 0.563, 0.071)	35	-	− 0.478 (− 0.983, 0.027)	0.005
Ultra-distal forearm	138	-	− 1.253 (− 1.820, − 0.686)	92	-	− 0.019 (− 0.714, 0.675)	38	-	0.764 (− 0.317, 1.844)	0.001
Mid-forearm	139	-	− 0.695 (− 1.029, − 0.361)	90	-	− 0.133 (− 0.549, 0.282)	38	-	− 1.136 (− 1.775, − 0.496)	0.021
HT users										
Femoral neck	67	-	0.003 (− 0.591, 0.598)	50	-	− 0.540 (− 1.228, 0.148)	12	-	− 1.074 (− 2.478, 0.331)	0.265
Lumbar spine	68	-	− 0.271 (− 0.688, 0.146)	50	-	− 0.149 (− 0.636, 0.337)	13	-	− 0.680 (− 1.634, 0.274)	0.619
Ultra-distal forearm	67	-	− 0.675 (− 1.264, − 0.086)	49	-	− 0.523 (− 1.212, 0.166)	12	-	− 0.179 (− 1.571, 1.213)	0.798
Mid-forearm	67	-	0.045 (− 0.448, 0.538)	50	-	− 0.017 (− 0.588, 0.553)	12	-	− 1.073 (− 2.238, 0.092)	0.211

Bold values indicate a statistically significant difference between groups

^a^Age presented as median (interquartile range)

^b^p value comparing the three time categories

^c^Values for age are the same as for “value per year” above

For HT users, there were no differences in rate of bone loss, either value or percentage, across the time categories.

### Bone Mineral Density Categories

**Fig. 1:**
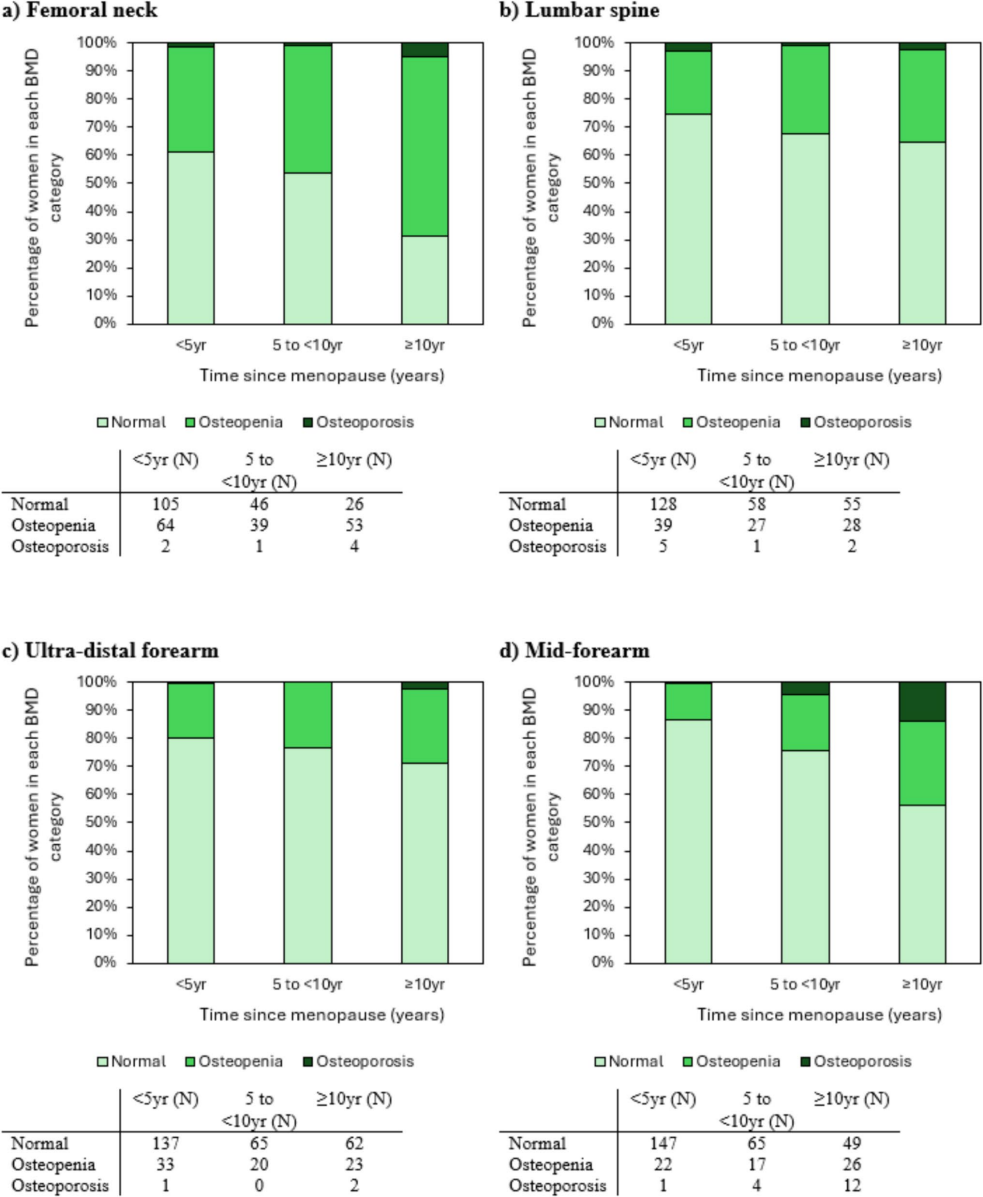
Proportions of non-users of hormone replacement therapy who had normal bone mineral density (BMD), osteopenia and osteoporosis at the (**a**) femoral neck, (**b**) lumbar spine, (**c**) ultra-distal forearm and (**d**) mid-forearm sites at different times since menopause (< 5 yr, 5 to < 10 yr and ≥ 10 yr). Number of participants in each time category are presented in tables below each sub-Figure

Figure 1 shows the proportions of HT non-users with normal BMD, osteopenia and osteoporosis across three time categories for time since menopause (< 5 yr, 5 to < 10 yr and ≥ 10 yr). The proportions of women with osteopenia and osteoporosis increased across the time categories at each skeletal site.

The increase in proportion of HT non-users with osteopenia and osteoporosis was greatest at the femoral neck and mid-forearm (p < 0.001). At the femoral neck the proportions of women with osteopenia increased across the time categories, from 37.4% to 45.3% to 63.9% at < 5 yr, 5 to < 10 yr and ≥ 10 yr, respectively. The proportions of women with osteoporosis also increased from 1.2% to 1.2% to 4.8% across the time categories. At the mid-forearm, the proportions of women with osteopenia across the time categories were 12.9%, 19.8% and 29.9%. The respective values for osteoporosis at the mid-forearm were 0.6%, 4.7% and 13.8%.

The increase in proportion of HT non-users with osteopenia and osteoporosis was less pronounced across the time categories for the lumbar spine and ultra-distal forearm compared to the other two sites (p = 0.330 and p = 0.368, respectively). At the lumbar spine, the proportion of women with osteopenia increased from 22.7% to 31.4% to 32.9% across the three time categories. The proportions of osteoporosis were; 2.9%, 1.2% and 2.4%, respectively. At the ultra-distal forearm, the proportion of women with osteopenia increased from 19.3% to 23.5% to 26.4% at the < 5 yr, 5 to < 10 yr and ≥ 10 yr time categories, respectively. The proportion of women with osteoporosis at the ultra-distal forearm was low across all the time categories; 0.6%, 0.0% and 2.3%.

**Fig. 2:**
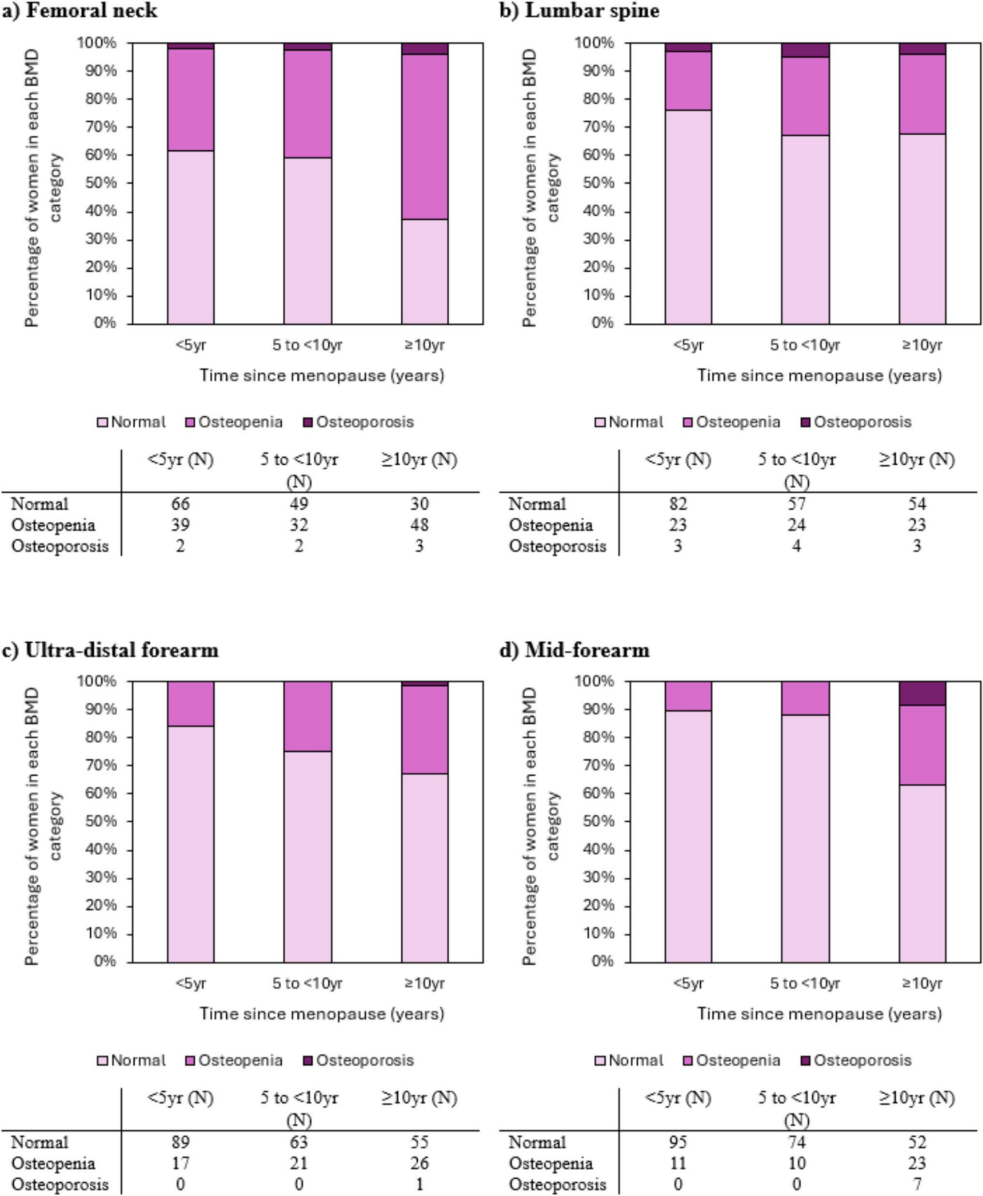
Proportions of users of hormone replacement therapy who had normal bone mineral density (BMD), osteopenia and osteoporosis at the (**a**) femoral neck, (**b**) lumbar spine, (**c**) ultra-distal forearm and (**d**) mid-forearm sites at different times since menopause (< 5 yr, 5 to < 10 yr and ≥ 10 yr). Number of participants in each time category are presented in tables below each sub-Figure

The results for HT users were similar (Fig. 2), where the increase in proportion of women with osteopenia and osteoporosis was more pronounced at the femoral neck and mid-forearm sites (p = 0.012 and p < 0.001, respectively). However, there was an increase in proportions of osteopenia and osteoporosis at the ultra-distal forearm (p = 0.035), but not the lumbar spine (p = 0.645).

Analyses were also performed for HT users and non-users combined, however, the results were similar to those for the stratified analyses (all p > 0.05) (Supplementary Fig. 2).

## Discussion

This study reported patterns of BMD loss following menopause at multiple skeletal sites. During the first five years postmenopause, the rate of bone loss was greater at the lumbar spine and ultra-distal forearm compared to the other two time categories. Bone loss was also more pronounced over time for non-users of HT compared to those who did use HT.

The Vietnam Osteoporosis Study has also reported patterns of femoral neck and lumbar spine BMD loss during the menopause transition [13]. A total of 1062 women were categorised into the following age groups: 40–44 years (premenopausal), 45–49 years (perimenopausal), 50–54 years (early postmenopausal), and 55–59 years (late postmenopausal) and had BMD measured twice, approximately two years apart. The study reported a small amount of bone loss occurred before the age of 45–49 years, followed by a greater decline between 50 and 54 years, which slowed between 55–59 years. Although the categorisation of menopause status was different in this study (based on age categories) compared to our study (based on self-reported menopause age), the results are similar, that a substantial amount of bone loss occurs during the first few years following menopause.

Another study from Iran examined the prevalence of osteopenia/osteoporosis at the femoral neck and lumbar spine among perimenopausal women (2.8 ± 1.5 years since menopause) with a mean age of 49.7 ± 2.0 years [14]. At the femoral neck, it was reported that 35.2% had osteopenia and 8.0% had osteoporosis. For the lumbar spine, the proportions of osteopenia and osteoporosis were 41.6% and 12.0%, respectively. The proportion of women with osteopenia at the femoral neck was comparable to that observed in our study for women within the group < 5 years since menopause (35.2% vs 37.1%). However, other proportions of osteopenia and osteoporosis were lower in our study. This may be due to differences in study population: our participants were population-based and not excluded on the basis of disease, while the Iranian study did have some exclusion criteria. Additionally, in our study, baseline T scores for the femoral neck were lower than at the other sites, which may explain the results. Despite this, both studies report a substantial proportion of women around the time of menopause to have osteopenia at the femoral neck or lumbar spine.

In this study, we reported that the rate of BMD loss was more pronounced during the first five years postmenopause at the sites with a higher proportion of trabecular bone, specifically the lumbar spine and ultra-distal forearm. This may be explained by bone turnover occurring faster in trabecular bone [4]. Other studies have also reported similar results, specifically a higher or similar rate of BMD loss at the lumbar spine, which is a primarily trabecular site, compared to the femoral neck, which contains a higher amount of cortical bone [3, 13, 15, 16]. Additionally, a study by Riggs et al. [9], which used quantitative computed tomography to assess rates of bone loss across the adult age range, reported that trabecular bone loss began in young adulthood, but cortical bone loss did not occur until older ages. Another study by Ó Breasail et al. [17] using peripheral quantitative computed tomography also reported similar results, showing that menopause was associated with lower bone density and strength at the distal radius; a site that contains a significant amount of trabecular bone.

In a longitudinal study by Greendale et al. [5], which included 705 pre- or perimenopausal women, examined changes in trabecular bone score (TBS), which is associated with trabecular microarchitecture, around the time of the FMP. The study reported that TBS began to decline 1.5 years prior to the FMP and this rate increased until approximately two years after the FMP, after which the rate of decline began to plateau.

Although bone loss is faster in trabecular bone, the majority of the skeleton is cortical (80%), and a previous study using high-resolution peripheral quantitative computed tomography (HR-pQCT) has shown that much of the bone remodelling in menopause occurs at sites with higher proportions of cortical bone [4]. The loss of trabecular bone at a specific location stops once the trabecular structure has been fully resorbed. However, for cortical bone, as more is resorbed, a larger surface area for further resorption is created [4]. These results together indicate that the loss of cortical bone during and shortly after menopause, in additional to trabecular bone loss, may be an important determinant of the risk of future fracture.

Another cross-sectional study has examined differences in pQCT-derived bone parameters among 430 women at different stages of menopause (pre-, peri- and post-menopause) [17]. The study reported that later menopause stage was associated with lower bone density and strength at the distal radius (4% site), as well as lower cortical density and thickness at the proximal radius (66% site). The reduction in cortical thickness observed for postmenopausal women supports the findings above, where cortical bone is resorbed, resulting in a reduced cortical thickness.

Previous studies have also examined risk factors for bone loss during the menopause transition. In a longitudinal study of southern Chinese women [16], lower age at menopause, higher baseline age, lower body weight and higher follicle-stimulating hormone concentration were associated with a higher BMD loss at the spine, femoral neck and total hip over a four year follow-up period. Similar results were reported in a Korean study [18] which showed that higher age, higher percent body fat, lower thyroid-stimulating hormone and lower serum uric acid levels were associated with a larger BMD loss at the lumbar spine over a two year follow-up period. Lower body weight and BMI as well as previous fragility fracture were also associated with a faster rate of BMD loss in another study [19] of 50 women aged < 60 years who were followed over 9 years. Targeting those risk factors which are modifiable may be useful in preventing or slowing bone loss during the menopause transition.

In Australia, bisphosphonates are government subsidised under specific conditions including age ≥ 70 years and a diagnosis of osteoporosis [20]. However, by the time a woman meets these criteria, they may have lost a substantial amount of bone and sustained one or more fractures. These women may have benefited from earlier preventative strategies, though additional work is needed to explore the cost-effectiveness and appropriateness of such an approach. It has previously been reported that HT use in postmenopausal women has been associated with a greater BMD and reduced non-vertebral fracture risk [21, 22], indicating a potential role for HT in the maintenance of bone health. The most recent position paper from The National Osteoporosis Guideline Group (NOGG) [23], recommends that HT should only be initiated for the treatment of post-menopausal osteoporosis in younger women (age ≤ 60 years) who have low risk for adverse malignant and thromboembolic events. Additionally, the 2022 version of the Hormone Therapy Position Statement of The North American Menopause Society (NAMS) [24] provides similar recommendations; for women aged < 60 years, within 10 years of menopause and no contraindications, HT has a favourable benefit-to-risk ratio for prevention of bone loss. It is also recommended that ongoing HT use should be discussed after the age of 60–65 years, taking into account the individual’s risks of adverse effects such as cancer, coronary heart disease, stroke, venous thromboembolism, and dementia [23, 24].

This study had several strengths and limitations. A major strength is that we had repeated measures of BMD at multiple sites over a long time period. We were also able to determine which participants were taking HT and stratify the analyses accordingly. Limitations include that menopause data were self-reported; hormone levels were not available at any of the follow-up assessment phases. HT use was updated at each follow-up, allowing ascertainment of the duration of use, however, this may have affected the results for the 5 to < 10 yr and ≥ 10 yr time categories. For example, a participant may have used HT during the first few years following menopause, and then stopped, which may have influenced bone loss during the later time periods post cessation. Additionally, subtle changes in participant positioning for the DXA scans, particularly for the forearm sites, could have occurred over the different assessment phases, which may have affected the resulting BMD measurements. However, at each assessment phase, positioning for the forearm scan was performed using a positioning board, and the participant was asked to make a loose fist with their hand. This was repeated systematically for each measurement, in an effort to reduce errors related to positioning.

## Conclusion

This study reported the pattern of BMD loss at multiple skeletal sites following menopause. A greater rate of bone loss occurred during the first five years postmenopause at the sites with a higher trabecular bone content, specifically the lumbar spine and ultra-distal forearm. Women who were taking HT experienced a less pronounced loss of BMD. A substantial proportion of women with recent menopause (< 5 years) had osteopenia at one or more of the skeletal sites examined. Future work will examine the relationship between BMD loss during and shortly following menopause and incident fracture.

## Supplementary Information

Below is the link to the electronic supplementary material.

Supplementary file1 (DOCX 244 KB)
