# Supplementary material for: Patterns of Bone Mineral Density Loss at Multiple Skeletal Sites Following Recent Menopause in Users and Non-Users of Menopausal Hormone Therapy
Source: Calcif Tissue Int. 2025 Jun 4;116(1):80. doi: 10.1007/s00223-025-01392-8 (PMC12137504; doi:10.1007/s00223-025-01392-8)
Supplement: Supplementary file 1 — Supplementary file1 (DOCX 244 KB) [file 223_2025_1392_MOESM1_ESM.docx]

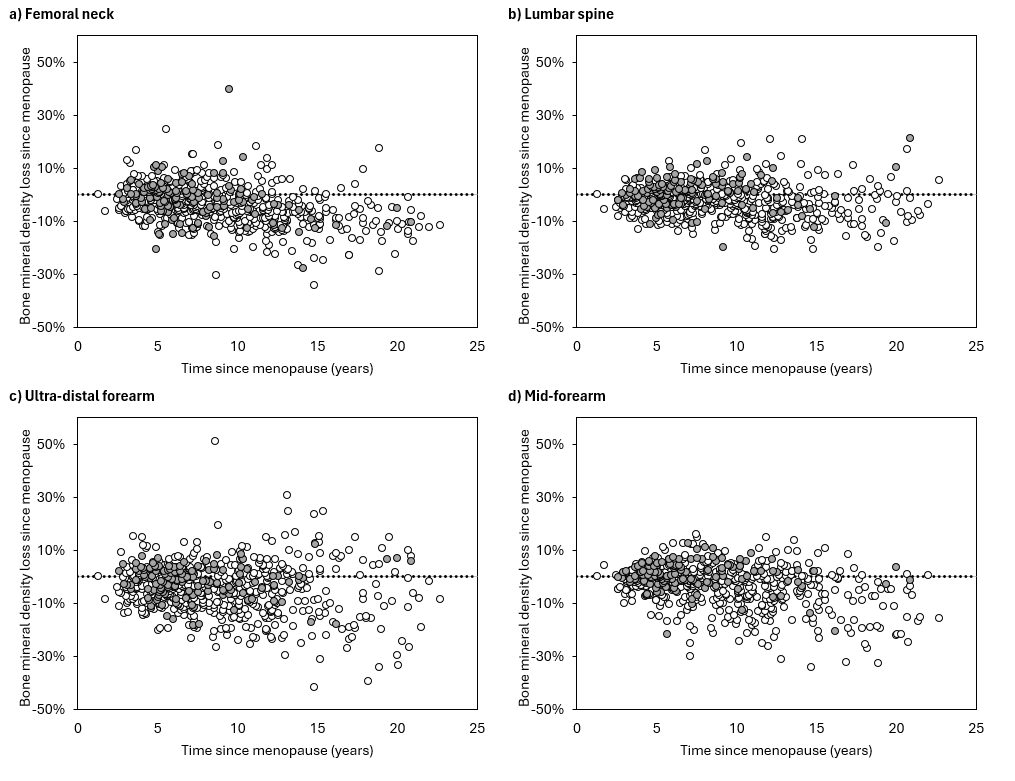


Supplementary Figure 1: Scatterplots showing time since menopause versus bone mineral density loss for the a) femoral neck, b) lumbar spine, c) ultra-distal forearm and d) mid-forearm sites. Light shading corresponds to hormone replacement therapy non-users and dark shading to hormone replacement therapy users.

| **a) Femoral neck**   \|  \| <5yr (N) \| 5 to <10yr (N) \| ≥10yr (N) \| \| --- \| --- \| --- \| --- \| \| Normal \| 171 \| 95 \| 56 \| \| Osteopenia \| 103 \| 71 \| 101 \| \| Osteoporosis \| 4 \| 3 \| 7 \| | **b) Lumbar spine**   \|  \| <5yr (N) \| 5 to <10yr (N) \| ≥10yr (N) \| \| --- \| --- \| --- \| --- \| \| Normal \| 210 \| 115 \| 109 \| \| Osteopenia \| 62 \| 51 \| 51 \| \| Osteoporosis \| 8 \| 5 \| 5 \| |
| --- | --- | --- | --- | --- | --- | --- | --- | --- | --- | --- | --- | --- | --- | --- | --- | --- | --- | --- | --- | --- | --- | --- | --- | --- | --- | --- | --- | --- | --- | --- | --- | --- | --- |
| **c) Ultra-distal forearm**   \|  \| <5yr (N) \| 5 to <10yr (N) \| ≥10yr (N) \| \| --- \| --- \| --- \| --- \| \| Normal \| 226 \| 128 \| 117 \| \| Osteopenia \| 50 \| 41 \| 49 \| \| Osteoporosis \| 1 \| 0 \| 3 \| | **d) Mid-forearm**   \|  \| <5yr (N) \| 5 to <10yr (N) \| ≥10yr (N) \| \| --- \| --- \| --- \| --- \| \| Normal \| 242 \| 139 \| 101 \| \| Osteopenia \| 33 \| 27 \| 49 \| \| Osteoporosis \| 1 \| 4 \| 19 \| |

Supplementary Figure 2: Proportions of users and non-users of hormone replacement therapy combined with normal bone mineral density (BMD), osteopenia and osteoporosis at the a) femoral neck, b) lumbar spine, c) ultra-distal forearm and d) mid-forearm sites at different times since menopause (<5yr, 5 to <10yr and ≥10yr).
